# Keeping a target in memory does not increase the effect of the Müller-Lyer illusion on saccades

**DOI:** 10.1007/s00221-015-4520-5

**Published:** 2015-12-21

**Authors:** Anouk J. de Brouwer, Eli Brenner, Jeroen B. J. Smeets

**Affiliations:** Department of Human Movement Sciences, Faculty of Behavioural and Movement Sciences, MOVE Research Institute Amsterdam, Vrije Universiteit Amsterdam, Van der Boechorststraat 7, 1081 BT Amsterdam, The Netherlands; Donders Institute for Brain, Cognition and Behaviour, Radboud University Nijmegen, Montessorilaan 3, 6525 HR Nijmegen, The Netherlands

**Keywords:** Gaze, Vision, Variability, Dorsal visual stream, Ventral visual stream

## Abstract

The effects of visual contextual illusions on motor behaviour vary largely between experimental conditions. Whereas it has often been reported that the effects of illusions on pointing and grasping are largest when the movement is performed some time after the stimulus has disappeared, the effect of a delay has hardly been studied for saccadic eye movements. In this experiment, participants viewed a briefly presented Müller-Lyer illusion with a target at its endpoint and made a saccade to the remembered position of this target after a delay of 0, 0.6, 1.2 or 1.8 s. We found that horizontal saccade amplitudes were shorter for the perceptually shorter than for the perceptually longer configuration of the illusion. Most importantly, although the delay clearly affected saccade amplitude, resulting in shorter saccades for longer delays, the illusion effect did not depend on the duration of the delay. We argue that visually guided and memory-guided saccades are likely based on a common visual representation.

## Introduction

Visual contextual illusions can affect our perception as well as our motor behaviour. The Müller-Lyer illusion, for example, changes the perceived length of a line segment through its inward or outward flanking arrowheads. This illusion can also affect the amplitude of pointing movements (e.g. Post and Welch 1996; de Grave et al. 2004) and saccadic eye movements (e.g. Binsted and Elliott 1999; de Grave et al. 2006), as well as the maximum grip aperture of grasping movements (Daprati and Gentilucci 1997; Franz et al. 2001), although it has been argued that the latter is not caused by the illusory size (Biegstraaten et al. 2007). The magnitude of the reported illusion effect varies largely between studies, depending on the experimental conditions. One of the factors that influence the effect is time. For instance, if the illusion is presented only briefly, its effect on saccade amplitude is larger than if it is presented for a longer time (van Zoest and Hunt 2011; Bertulis et al. 2014; de Brouwer et al. 2014). For grasping, it has also been suggested that a longer preview of the Müller-Lyer illusion induces smaller effects on maximum grip aperture (Bruno and Franz 2009). In the present study, we will focus on a second temporal factor: the delay between the stimulus disappearance and the execution of the response.

The largest effects of visual contextual illusions on pointing and grasping seem to occur when the movement is performed after a delay during which the stimulus is not visible (Elliott and Lee 1995; Gentilucci et al. 1996; Westwood et al. 2000; Gentilucci et al. 2001; Westwood et al. 2001; Rival et al. 2003; Brownell et al. 2010) (but see Glazebrook et al. 2005; Mendoza et al. 2005). Several researchers have suggested that these effects occur because memory-guided movements rely on a different representation than visually guided movements (Goodale and Milner 1992; Hu and Goodale 2000). Specifically, these authors argue that memory-guided movements are based on the processing of visual information into viewer-invariant (i.e. allocentric) representations that are suited for long-term storage, a process that takes place in the ventral visual stream of the brain. They further argue that because allocentric representations take visual context into account, these representations are highly sensitive to visual contextual illusions. In contrast, for visually guided movements the position of the target must be specified on a moment-to-moment basis in egocentric coordinates, that is, with respect to the observer. In the dorsal visual stream that mediates this process, the target position can therefore be specified independent of the context. Thus, visually guided movements should be insensitive to visual illusions (Milner and Goodale 2006). Because egocentric information decays rapidly when the target disappears from vision, an allocentric representation—that is assumed to be highly sensitive to visual illusions—is used for memory-guided movements. Whereas it was initially suggested that egocentric information may exist up to 2 s (Elliott and Madalena 1987), more recent studies have revealed much quicker decays of egocentric information (Rosetti et al. 1994 as shown in Fig. 4.11 of Rossetti and Pisella 2002; Westwood and Goodale 2003; Goodale et al. 2004; Rolheiser et al. 2006; Hesse and Franz 2010).

For saccades, however, the increase in illusion effect in memory conditions has not been reported, neither has it been rigorously studied. It has been suggested that there is no difference in effects of the Müller-Lyer illusion on saccades in response to the appearance of the illusion and voluntary saccades (including memory-guided and deferred saccades) (Bruno et al. 2010). In agreement with this suggestion, in a recent experimental study we did not find a difference in illusion effect between memory-guided (0.8-s delay) and visually guided saccades to a briefly presented Müller-Lyer illusion (de Brouwer et al. 2014). Knox and Bruno (2007) even found a smaller effect of this illusion on saccade amplitude to remembered (2-s delay) than to visual targets. These results are inconsistent with the idea that there is a shift from an egocentric representation that is insensitive to visual illusions to an allocentric representation that is highly affected by illusions.

Without the context of illusions, saccades are clearly affected by a delay during which the target is not visible: memory-guided saccades show larger systematic errors and more variability in endpoints than do saccades to visual targets (Gnadt et al. 1991; White et al. 1994; Rolheiser et al. 2006). This suggests that there is a shift from a relatively accurate egocentric representation for visible targets to a less accurate allocentric representation for remembered targets (Gnadt et al. 1991; Rolheiser et al. 2006). The moment at which this shift would occur is not entirely clear. Most of the systematic errors in saccade endpoints have been found to accumulate within 1 s of delay (Gnadt et al. 1991; White et al. 1994). Whereas both Gnadt and colleagues and Rolheiser and colleagues reported a steep initial increase in endpoint variability within 0.5–1 s of delay, White and colleagues reported that the variability increases monotonically up to delays of several seconds. In any case, the endpoints of memory-guided saccades should be less accurate and more variable than those of saccades to visual targets, even after brief (≤1 s) delays.

The experimental findings on saccade endpoint variability in memory conditions thus suggest a shift from an egocentric to an allocentric target representation, whereas the absence of an increase in illusion effects on memory-guided saccades suggests that the same representation is used independent of any delay. The aim of this study is therefore to determine the influence of delays of various durations on the effect of the Müller-Lyer illusion on saccades as well as the effect of delays on saccade endpoint variability.

## Methods

### Participants

Eighteen participants (aged 19–24, six men) took part in the experiment after providing their informed consent. One participant was removed from the analysis because he only performed 55 % of the trials correctly (compared to 68–96 % for the other participants, see “Data analysis” section for criteria). Most trials were rejected because this participant showed a large error (>5°) in the initial fixation position, which was probably due to a calibration error. All participants had normal or corrected-to-normal vision. The study was part of a research programme that was approved by the local ethics committee (Faculty of Behavioural and Movement Sciences, Vrije Universiteit Amsterdam, The Netherlands) and was performed in accordance with the ethical standards laid down in the 1964 Declaration of Helsinki.

### Set-up

Participants were seated in a dimly lit room, with their head stabilized by a chin rest positioned about 52 cm from a computer screen (36 × 27 cm, 1024 × 768 pixels, 85 Hz refresh rate). At this distance, 1.0 cm on the screen corresponds to approximately 1.1° of visual angle. Visual stimuli were controlled using the Psychophysics toolbox (Brainard 1997) for MATLAB (MathWorks Ltd., USA). Eye movements of both eyes were recorded with an Eyelink II eye tracker (SR Research Ltd., Canada), with a temporal resolution of 500 Hz and a spatial accuracy of <0.5° of visual angle.

### Stimuli

**Fig. 1:**
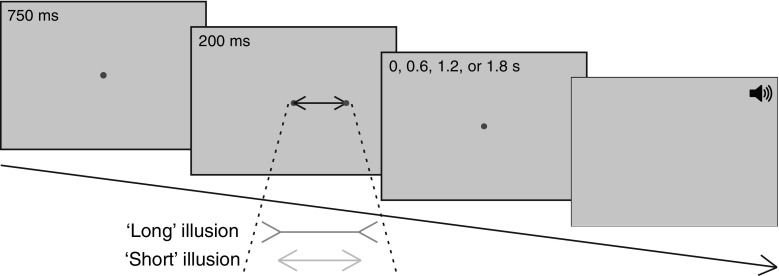
Schematic illustration of the task. Participants had to fixate the dot at the centre of the screen and remember the position of the target dot on a briefly presented (200 ms) Müller-Lyer illusion while maintaining fixation. After a delay of 0, 0.6, 1.2 or 1.8 s, the fixation dot disappeared and a brief tone sounded to cue the participant to make a saccade to the remembered target position

The stimulus was a horizontal Müller-Lyer illusion with a shaft length of 5.5° or 7.7° of visual angle (5.0 or 7.0 cm) and a red target dot at one of its endpoints. Fins of 1.7° or 2.3° of visual angle (30 % of shaft length) were attached to the horizontal shaft with an angle of 150° (‘long’ illusion) or 30° (‘short’ illusion, see Fig. 1). The illusion was drawn in black lines of about 0.1° thick on a light grey background. One end of the shaft appeared at a blue fixation dot that was presented at the centre of the screen, and the other end was marked with a red target dot. Both dots had a diameter of 0.4°. Two shaft lengths and two saccade directions (left and right) were used to prevent participants from planning a standardized response.

### Procedure

Figure 1 shows a schematic illustration of the task. Each trial started with the presentation of the fixation dot at the centre of the screen for 750 ms. Then, the stimulus (the Müller-Lyer illusion with the target dot) was presented to the left or right of the screen centre for 200 ms, while the fixation dot remained visible. After a delay of 0, 0.6, 1.2 or 1.8 s in which the stimulus was not visible, the fixation dot disappeared and a 50-ms tone sounded as a cue to make a saccade. Participants were instructed to maintain fixation during the delay and to move their eyes to the remembered position of the target when the cue was presented. A new trial started 1.7 s after the cue.

The experiment contained 32 different conditions: 2 fin configurations × 2 shaft lengths × 2 directions × 4 delays. After 32 practice trials (one for each condition), participants performed four runs of four repetitions of each condition (32 × 4 = 128 trials per run), with short breaks in between. Trials were presented in randomized sets of one repetition of each condition. If the participant made a saccade towards the illusion before the cue, a red bar was presented at the centre of the screen to indicate that the participant had made an error. These trials were repeated at the end of the run.

### Data analysis

The eye positions given by the eye tracker were averaged across the left and right eye and used to calculate horizontal and vertical eye velocities. The resultant velocity was used to define saccade onset and offset. Saccades were identified by having a peak velocity above 75°/s for two or more (≥4 ms) consecutive samples. Saccade onset was defined as the last of five consecutive samples (10 ms) before eye velocity reached a 30°/s threshold preceding the velocity peak. Saccade offset was defined as the first of five consecutive samples (10 ms) below the 30°/s threshold after the velocity peak. Saccades with an amplitude of 2.0° or more were analysed. Trials were discarded if the pupil was not tracked during the whole duration of the saccade, or if the saccade onset occurred before the cue. Drift of the eye tracker within 5° was corrected for by assuming correct fixation during the onset of the stimulus. These fixations were calculated as the mean eye position during 10 consecutive samples (20 ms) in which eye velocity was below the 30°/s threshold within a window of 200 ms surrounding target onset. Trials were discarded if the eyes were moving too fast within this time window, if the correction was larger than 5°, or if the eye drifted further than 1° from the fixation position before saccade onset. Further, trials were discarded if the saccade did not move the eyes closer to the target, or if the saccade ended more than 2.0° vertically from the target position.

For the correct trials, we calculated the median horizontal saccade amplitude for each of the 32 conditions. Saccade amplitude was defined as the distance between the eye position at saccade offset and the eye position during fixation (averaged across 20 ms while fixating, as explained above). The absolute horizontal amplitudes were averaged over saccade direction (left and right). A 2 × 2 × 4 repeated measures ANOVA with the within-subject factors illusion (‘short’ and ‘long’), shaft length (5.5° and 7.7°) and delay (0, 0.6, 1.2 and 1.8 s) was performed on the amplitudes. Since our main question is whether the duration of the delay influences the size of the illusion effect, we were particularly interested in a possible illusion × delay interaction effect.

To investigate whether the duration of the delay influences the variability in horizontal saccade amplitude, we calculated the interquartile range of amplitudes in each condition. The interquartile range describes the width of the middle 50 % of the distribution of amplitudes. This was calculated for all participants who had a minimum of 10 (out of 16) correct saccades in all 32 conditions. The interquartile ranges were averaged over saccade direction (left and right). A 2 × 2 × 4 repeated measures ANOVA with the within-subject factors illusion (‘short’ and long’), shaft length (5.5° and 7.7°) and delay (0, 0.6, 1.2 and 1.8 s) was performed on the interquartile ranges. Here, we were particularly interested in an effect of delay on the interquartile ranges. For all statistical tests, a significance level of 0.05 was used. When the assumption of sphericity was violated, the Greenhouse–Geisser correction was used.

## Results

**Fig. 2:**
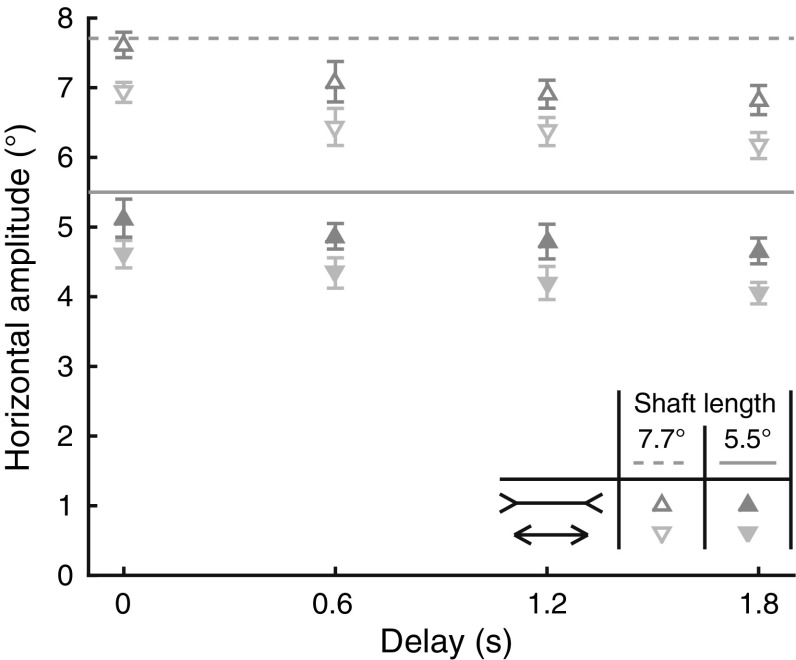
Horizontal saccade amplitude for the two shaft lengths and illusions as a function of the duration of the delay following the disappearance of the illusion. *Error bars* depict the standard errors across participants (*n* = 17)

We investigated the influence of the duration of a delay, ranging from 0 to 1.8 s, on the effect of a briefly presented Müller-Lyer illusion on saccade amplitude. Figure 2 shows the horizontal saccade amplitude for both shaft lengths and both configurations of the illusion as a function of the duration of the delay. Obviously, saccades were shorter for the smaller shaft lengths [*F*(1,16) = 693.7, *p* < 0.001]. Saccade amplitudes were influenced by the illusion; they were on average 0.6° longer for the ‘long’ illusion than for the ‘short’ illusion [*F*(1,16) = 179.0, *p* < 0.001]. Further, saccade amplitudes decreased with longer delays [*F*(1.5,24.2) = 34.4, *p* < 0.001, Greenhouse–Geisser corrected]. The ANOVA also showed a significant shaft length × delay interaction effect [*F*(3,48) = 3.6, *p* = 0.019]: the decrease in saccade amplitudes was somewhat more pronounced for the smaller shaft lengths. Most importantly, the illusion × delay interaction was not significant [*F*(3,48) = 0.2, *p* = 0.909], showing that the size of the illusion effect did not significantly change with different delays. Thus, saccades were shorter for increasing delay durations, but the effect of the Müller-Lyer illusion was not influenced by the duration of the delay.

**Fig. 3:**
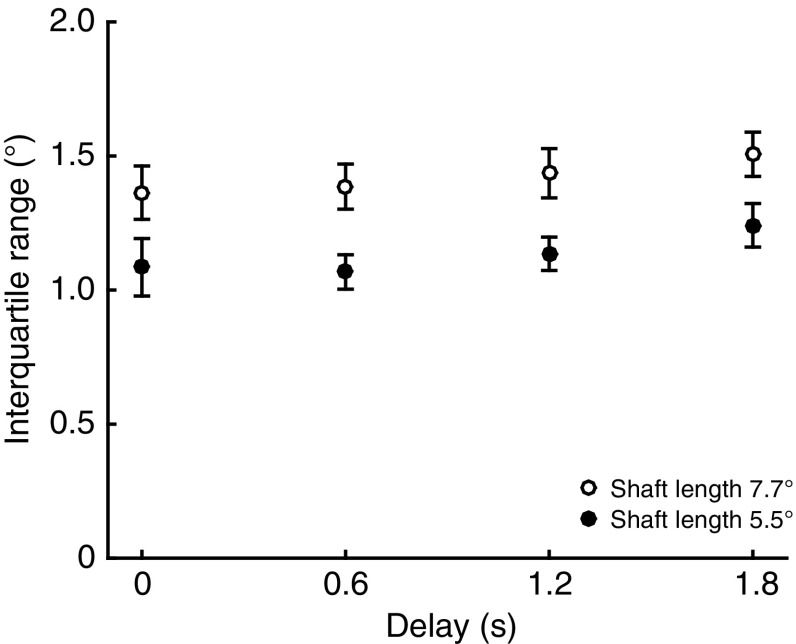
Interquartile range of horizontal saccade amplitudes for the two shaft lengths as a function of the duration of the delay following the disappearance of the illusion. The interquartile ranges were averaged across the ‘long’ and ‘short’ illusions. *Error bars* depict the standard error across participants (*n* = 14)

We also investigated whether the variability in horizontal saccade amplitude is influenced by the duration of the delay. Figure 3 shows the interquartile ranges averaged across the 14 participants that had at least 10 correct saccades in each condition. Interquartile ranges were significantly larger for the greater shaft lengths [*F*(1,13) = 42.4, *p* < 0.001]. Although there appears to be a slight increase in the size of the interquartile range for longer delays, this effect was not significant [*F*(2.1,27.3) = 2.881, *p* = 0.071, Greenhouse–Geisser corrected]. The configuration of the illusion did not affect the interquartile ranges [*F*(1,13) = 0.2, *p* = 0.644], and there were no significant interaction effects. These results show that the variability in horizontal saccade amplitude does not increase with longer delays.

## Discussion

It has often been reported that the largest effects of visual contextual illusions on pointing and grasping occur when the movement is performed after a delay in which the illusion is not visible. In the present study, we showed that this is not true for the effects of illusions on saccadic eye movements. Eighteen participants viewed a briefly presented Müller-Lyer illusion with a target at its endpoint and performed a saccade to the (remembered) position of this target after a delay of 0, 0.6, 1.2 or 1.8 s. The horizontal saccade amplitudes showed a clear effect of the illusion: they were on average 0.6° shorter for the perceptually short illusion than for the perceptually long illusion. Saccades became shorter when the duration of the delay increased, but the delay did not influence the size of the illusion effect. Further, we found that the variability in saccade amplitudes was not affected by the duration of the delay.

Expressed as a percentage of saccade amplitude, the illusion effects that we found in the current study ranged from 10 to 12 % (mean ± SEM 10 ± 1, 11 ± 1, 11 ± 1 and 12 ± 1 % for the 0-, 0.6-, 1.2- and 1.8-s delay, respectively). This is very similar to the size of the illusion effect we reported for a briefly presented Müller-Lyer illusion in a previous study (de Brouwer et al. 2014). In the present study, we did not find an increase in the illusion effect when the duration of the delay increased from 0 to 1.8 s. Our results confirm the suggestion that there is no difference in illusion effect on reflexive and memory-guided saccades (Bruno et al. 2010). On the other hand, our findings are inconsistent with the hypothesis that visually guided movements and memory-guided movements rely on different visual representations, causing memory-guided movements to be more affected by illusions than visually guided movements. When discussing the present results, one could argue that our 0-s delay condition is not a visually guided condition. The reason would be that in this condition the response was cued at the moment the illusion and target disappeared. According to the view of real-time control of action, visuomotor mechanisms in the dorsal visual stream are only engaged if the target is visible when the response is cued (Goodale et al. 2004). This view was based on the finding that grasping movements became sensitive to an illusion when vision was removed at the moment the cue to respond was given (i.e. 0-s delay) while grasping was not affected in a full-vision condition (Westwood et al. 2000; Westwood and Goodale 2003; Brownell et al. 2010). However, in a previous study we already showed that for a briefly presented Müller-Lyer stimulus (153 ms), the illusion effect does not differ between visually guided saccades and saccades performed after a delay (Experiment 1A in de Brouwer et al. 2014). Further, one could argue that the delays used in the present study are not sufficiently long for the egocentric information to decay. However, we do not consider this possibility very likely. Several studies have shown decreased accuracy and increased variability in saccade endpoints within delays of 1 s (Gnadt et al. 1991; White et al. 1994; Rolheiser et al. 2006), suggesting that there is a quick decay of the egocentric representation. In accordance with this observation, we found an increase in systematic error (Harris 1995) for longer delay durations, although the time course that we observed was more gradual than in previous studies. In contrast to previous studies, we did not find an increase in the variability in horizontal saccade amplitude with longer delays. Together, the results of the present experiment show that the effect of the Müller-Lyer illusion is not influenced by the time between the disappearance of the illusion and the cue to execute the saccade.

How can we reconcile our results with the often-reported finding that visually guided pointing and grasping are hardly affected by illusions, whereas these movements are largely affected by illusions when they are performed after a delay? A possible explanation is provided by Franz and colleagues (2009), who proposed that visual feedback plays a critical role in how illusions affect hand movements. Their idea is that under conditions where vision of the target and hand is available during the movement, feedback mechanisms could detect the error introduced by the illusion and allow one to perform online corrections. When comparing a visually guided condition to a memory-guided condition, there is a confound between memory demands and the availability of visual feedback during the execution of the movement. In their experiment, Franz and colleagues replicated the increase in the illusion effect when introducing a delay between the presentation of the illusion and the grasping movement. Critically, they found that the illusion had the same effect in a condition where vision of the target and hand was removed during the movement as in a condition where the movement was executed after a 5-s delay. Hence, they concluded that illusion effects depend strongly on the availability of visual feedback, not on the use of different visual representations. As saccades are typically too brief to allow for online corrections, our results showing that a delay does not influence the illusion effect on saccades are in line with their explanation.

Another possible explanation for the different influence of delays on how illusions affect saccades and hand movements is that these two kinds of movements are based on different information. It is likely that saccades are programmed in terms of a distance from the current gaze position to the target, and this perceived distance is affected by the Müller-Lyer illusion. The grip aperture in visually guided grasping movements is probably based on the positions of the individual digits’ contact points, so errors in the perceived distance between them due to the illusion are irrelevant (Smeets and Brenner 1999; Biegstraaten et al. 2007). With a delay, these egocentric positions become less certain, so it becomes advantageous to rely on memory of the (misjudged) size of the object to guide the movement (Hu and Goodale 2000; Smeets and Brenner 2008). As a result, illusion effects on grasping increase with a delay, whereas the effects on saccades do not.

## Conclusion

We used the Müller-Lyer illusion in a saccade task with delays of 0, 0.6, 1.2 and 1.8 s between the disappearance of the stimulus and the cue to respond. Saccade amplitude was affected by the illusion, but the size of this effect did not depend on the duration of the delay. Further, the variability in saccade amplitudes was not affected by the duration of the delay. The lack of increase in illusion effect when a delay is introduced suggests that it is unlikely that visually guided and memory-guided saccades are based on different visual representations.
